# Premature progesterone rise at human chorionic gonadotropin triggering day has no correlation with intracytoplasmic sperm injection outcome

**Published:** 2015-02

**Authors:** Nasrin Saharkhiz, Saghar Salehpour, Mahboobeh Tavasoli, Ali Aghighi

**Affiliations:** 1 *Infertility and Reproductive Health Research Center (IRHRC), Shahid Beheshti University of Medical Sciences, Tehran, Iran.*; 2 *Baqiyatallah University of Medical Sciences, Tehran, Iran.*

**Keywords:** *Progesterone rise*, *HCG triggering*, *GnRh agonist*, *GnRh antagonist*, *Intracytoplasmic sperm injection*

## Abstract

**Background:**

Premature luteinization during in vitro fertilization was commonly happened before the introduction of GnRh analogues. High level of unwanted progesterone is associated with adverse pregnancy outcome and is thought to be induced by inappropriate LH elevation.

**Objective:**

To evaluate the progesterone level on the day of Human Chorionic Gonadotropin (HCG) triggering in GnRh agonist and antagonist protocols, and its correlation with clinical pregnancy rate and miscarriage rate.

**Materials and Methods:**

One hundred and seven women underwent intracytoplasmic sperm injection with long agonist protocol (n=46) or antagonist protocol (n=61). Blood sample was obtained from each patient for progesterone level measurement in HCG administration day, then patients were divided into two groups according to their serum progesterone levels on the HCG triggering day: progesterone level <1.2 ng/ml, and progesterone level ≥1.2 ng/ml. Clinical pregnancy and miscarriage rates were evaluated as main outcomes and biochemical pregnancy rate and implantation rate were considered as secondary outcomes.

**Results:**

The increased prevalence rate of premature progesterone (progesterone level ≥1.2 ng/ml) in total patients was 13.1% (14/107) and in long agonist protocol group and antagonist protocol group was 15.2% (7/46) and 11.5% (7/61) respectively. Premature progesterone rise had no significant correlation with clinical pregnancy rate in total patients (p=0.174), agonist protocol (p=0.545), and antagonist protocol (p=0.129). Also premature progesterone rise had no significant association with miscarriage rate in total patients (p=0.077), agonist protocol group (p=0.383) and antagonist protocol group (p=0.087).

**Conclusion:**

A significant rise in progesterone levels at the time of HCG triggering doesn’t lead to decrease in pregnancy rate and implantation rate and increase in miscarriage rate.

## Introduction

Premature luteinization in in vitro fertilization (IVF) was a common event before the introduction of Gonadotropin-releasing hormone (GnRH) analogues (1). This situation associated with poor oocyte quality, low fertilization rate, and adverse pregnancy outcome and is thought to be induced by inappropriate Luteinizing hormone (LH) elevation (2). During controlled ovarian hyperstimulation premature luteinization, as detected by elevated serum progesterone (P) level, is generally prevented by suppression of LH secretion with GnRh analogues (3). However in the majority of cases, despite the use of GnRH analogues, a significant rise in serum P levels is observed in a subgroup of women, particularly at the end of the stimulation cycle (1). Incidence of premature progesterone rising (PPR) has high levels approximately 35% in GnRH agonist cycles and 38% in GnRH antagonist cycles (4-8). 

Hence PPR must to be given more attention. Many papers have been published about PPR. In some cases, PPR has associated with decreased pregnancy rate, but some others have shown positive effect of PPR on pregnancy rate (8-13). There are some reports that have indicated PPR has no effect on pregnancy rate (14-16). In an analysis included more than 4,000 cycles, Pregnancy rate and implantation rate were significantly lower in the premature luteinization group and especially in the group with progesterone level more than 1.5 ng/mL (8). The underlying mechanism that leads to lower pregnancy rates is unclear (4).

During controlled ovarian hyper-stimulation (COH), excessive follicular development and supraphysiologic serum concentrations of E_2_ can lead to a premature rise of P in the late follicular phase which resulting in asynchrony associated with implantation failure (8, 17, 18). Additional hypotheses have dealt with different factors involved in pregnancy such as insufficient function of LH and FSH for suppression and desensitization of receptors, serum accumulation of hCG from human menopausal gonadotropin )hMG(, increased LH receptor sensitivity of the granulosa cells, poor ovarian response with increased LH sensitivity, disruption of signaling in the ovarian granulose cells, and also decreased endometrial receptivity can lead to decreased live birth rate (1, 19-25). It is clinically relevant to address these questions, because if PPR is harmful to clinical outcomes, either triggering HCG in advance or freezing all embryos should be recommended to avoid the adverse consequences (1).

We hypothesized that a subtle rise in progesterone levels at the time of HCG triggering may lower the pregnancy rate. Accordingly, in the present study, we described both the distribution of progesterone on the day of triggering in women who had agonist protocol or antagonist protocol and the correlation between premature rising of progesterone level and pregnancy outcomes in agonist and antagonist protocols.

## Materials and methods

**Under-study population and design**

This study has been approved by the Infertility and Reproductive Health Research Center (IRHRC), Shahid Beheshti University of Medical Sciences, Tehran, Iran and was approved by the Ethical Committee of Shahid Beheshti University of Medical Sciences. An informed consent was obtained from each participant. This nonintervention, prospective, single-center cohort study was performed on the unfertilized women have been referred to Taleghani Infertility and IVF Center to seek medical treatment between 20 January 2013 and 20 January 2014. 

All women in this study were started their ICSI cycles with GnRh agonist or antagonist protocol in the IVF center. Criteria for inclusion were female age <40 years and basal FSH <10 mIU/mL. Participants underwent COH with rFSH (Gonal-F, Merck, Serono) or highly purified hFSH (Fostimon, IBSA).The progesterone level was measured on the day of HCG triggering. Only patients who had completed the IVF/ICSI-embryo transfer (ET) cycle and had blood test in Taleghani Hospital Laboratory were included. 

Women who had a blood test either from another laboratory or on any other day than those outlined above or had not fresh embryo transfer were excluded. Using flexible protocol GnRH antagonist (Cetrorelix, Cetrotide, 0.25 mg/d SC; Merck Serono) was started when at least one leading follicle reached 13-14 mm in size and considerably, oral contraceptives were not used for pretreatment. Antagonist protocol was used for women in the older age group and polycystic ovarian syndrome (PCOS) group. In long agonist protocol, oral contraceptive (low dose) was used for pretreatment and buserelin acetate (Suprefact, Sanofi, SQ) was started for at least 7 days before the onset of ovarian stimulation. 

The dose of gonadotropin was individualized for each patient based on the age, anti mullerian hormone (AMH) level, previous response to ovarian stimulation and FSH level on the third day of menstruation within maximum 6 months ago. In addition, antral follicles were counted by ultrasound test on day 3 of menstrual cycle to evaluate ovarian reserve. Gonadotropin was started on day 2-3 of menstrual cycle. When at least two follicles reached a mean diameter of 18 mm, 10,000 IU of hCG (Choriomon, IBSA) were injected directly into muscle and the oocytes were retrieved by trans-vaginal ultrasound guided aspiration 36 hr later. 

All embryos transferred were 6 cells or greater and were of A or B quality with a maximum of three embryos. For the purpose of luteal support, micronized progesterone (Cyclogest suppository; Actavis) at a dose of 400 mg/bid was applied vaginally from embryo transfer day for at least 14 days after oocyte retrieval. Luteal support was continued until 12 weeks of gestational age for those who had gotten pregnant. Oocytes were fertilized in vitro by ICSI. All embryo transfers where carried out after 2-3 days of culture. Serum β-hCG levels were measured two times (14 and 16 days after embryo transfer). In the cases that β-hCG was positive, trans-vaginal sonography was performed at 7^th^ week of gestation.

**Hormone assays**

Blood sample was obtained at the day of HCG at 9-10 am for P level measurement and analyzed by radioimmunoassay with a sensitivity of 0.2 ng/ml (range of measurement was 0.2-40 ng/ml). The within-assay variability was 7-10%.

**Primary and secondary outcomes**

The primary outcomes were clinical pregnancy rate and miscarriage rate. The secondary outcomes were biochemical pregnancy rate and implantation rate. Biochemical pregnancy was defined as having a positive β-hCG approximately 14-16 days after embryo transfer. Clinical pregnancy was defined as report of fetal heart beat in ultrasound at 5 weeks after embryo transfer. Implantation rate was defined as the number of gestational sac seen in ultrasound divided by the total number of transferred embryos.

**Statistical analysis**

Patients were divided into two groups according to their serum P levels on the HCG triggering day: p<1.2 ng/ml, and p≥1.2 ng/ml. All cycle characteristics were reported as the mean±SD. Statistical analysis was performed with the Statistical Package for Social Sciences (SPSS 16.0 for Windows). P<0.05 was considered to be statistically significant. Independent Student’s *t* test, ^2^- test, and Fisher’s exact test were used for data analysis. The correlation between serum P level in two groups on the day of hCG administration, biochemical and clinical pregnancy, as same as implantation and miscarriage rates were analyzed by Spearman correlation coefficient. Then this analysis was performed for other two groups: p<1.5 ng/ml, and p≥1.5 ng/ml.

## Results

Among total number of 122 women, 107 women completed an ICSI-ET cycle during the study period and were included in the final analysis. Fifteen patients had not the final inclusion criteria because they either did not performed serum P test on the HCG triggering day or they had taken the P test in another laboratory (n=5) or had freeze oocyte (n=2), or they did not undergo immediate ET (n=8) because of ovarian hyperstimulation syndrome risk of endometrial polyp. 

The primary or combined indications for intracytoplasmic sperm injection were male subfertility n=45 (42.05%), polycystic ovarian syndrome n=27 (25.23%), tubal pathology n=16 (14.95%), endometriosis n=7 (6.54%), and other causes n=12 (11.21%). Baseline characteristics of study participants are shown in table I. In total 46 (43%) patients were in long agonist protocol group and 61 ones (57%) in antagonist protocol group. The mean age (agonist group: 29.30 vs. antagonist group: 34.25, p<0.001) and infertility duration (agonist group: 5.83 vs. antagonist group: 7.77, p=0.044) between agonist and antagonist groups were significantly different. There was not any case of ectopic pregnancy. The mean of the P level on the HCG triggering day was 0.75±0.49 in total patients and 0.81±0.54 in agonist group and 0.81±0.54 ng/ml in antagonist group and there were no significant differences between two groups (p=0.192) (Table I). 

Overall 93 patients (86.91%) were in p<1.2 ng/ml group and 14 (13.08%) were in p≥1.2 ng/ml group. Age of two groups, antral follicle count, total number of embryos and number of frozen embryos were not significantly different. Elevated P level was not associated with either lower biochemical pregnancy rate, clinical pregnancy rate, implantation rate or higher miscarriage rate (Table II). P levels on day of HCG triggering was not inversely associated with clinical pregnancy rate in agonist protocol (p=0.545) and antagonist protocol (p=0.129). PPR had no significant correlation with biochemical pregnancy rate in agonist protocol (p=0.677) and antagonist protocol (p=0.530). PPR had no significant correlation with miscarriage rate in agonist protocol (p=0.383) and antagonist protocol (p=0.087) and finally miscarriage rate was not significantly associated with p≥1.2 in agonist protocol (p=0.970) and antagonist protocol (p=0.113) (Table III). 

The incidence of p≥1.5 ng/ml in total patients was 2.8% (3/107) and in long agonist protocol and antagonist protocol was 4.3% (2/46) and1.6% (1/61) respectively. This P level had no significant correlation with clinical pregnancy rate in total patients (p=0.897) and agonist protocol (p=0.754) and antagonist protocol (p=0. 589).No one conceived with a p≥1.5 ng/ml (Table IV).

**Table I:** Baseline and cycle characteristic of total patients and long agonist and antagonist groups

**Characteristic**	**Total patients**	**Agonist group**	**Antagonist group**	**p-value**
Age (y)	32.12 ± 5.90	29.30 ± 4.13	34.25 ± 6.18	0.000^a^
Infertility duration (y)	6.90 ± 5.21	5.83 ± 3.91	7.77 ± 5.92	0.044^a^
Body mass index (kg/m^2^)	24.68 ± 4.81	24.28 ± 5.39	24.98 ± 4.34	0.458^a^
Antral follicle count )n)	10.48 ± 4.39	10.76 ± 4.12	10.26 ± 4.60	0.564^a^
rFSH total dose administered (U)	2082 ± 845.85	1990.5 ± 872.25	2150.25 ± 832.5	0.336^a^
Duration of stimulation (day)	8.96 ± 2.07	9.11 ± 1.91	8.85 ± 2.20	0.530^a^
progesterone level on D HCG (ng/ml)	0.75 ± 0.49	0.81 ± 0.54	0.71 ± 0.36	0.192^a^
NO of oocytes retrieved	7.26 ± 5.47	7.70 ± 5.38	6.93 ± 5.55	0.479^a^
Total number of embryos	4.57 ± 4.41	4.63 ± 3.95	4.52 ± 4.76	0.883^a^
No of embryos frozen	2.49 ± 4.05	2.57 ± 3.87	2.43 ± 4.26	0.861^a^
Clinical pregnancy rate (%)	32/107 (29.9)	18/46 (39.13)	14/61 (22.95)	0.032^b^
Biochemical pregnancy rate (%)	36/107 (33.64)	19/46 (41.30)	17/61 (27.86)	0.042^b^
Implantation rate (%)	38/209 (18.18)	22/94 (23.40)	16/115 (13.91)	0.034^b^
Miscarriage rate (%)	8/36 (22.22)	1/19 (5.26)	7/17 (41.17)	0.007^c^

D HCG=day of hCG administration

a Independent Student’s *t* test

b χ^2^- test

c Fisher’s exact test

**Table II:** Characteristics of participants stratified by progesterone (P) level on day of HCG

**Characteristic**	**p≥1.2, n=14**	**p<1.2 , n=93**	**p-value**
Age (y)	32.57 ± 6.24	32.05 ± 5.88	0.762^a^
NO of Antral follicle count	10.29 ± 5.34	10.51 ± 4.26	0.863^a^
NO of oocytes retrieved	6.79 ± 4.99	7.33 ± 5.56	0.729^a^
Total number of embryos	4.07 ± 4.41	4.66 ± 4.42	0.646^a^
NO of frozen embryos	2.43 ± 4.07	2.49 ± 4.06	0.955^a^
Biochemical pregnancy rate (%)	2/14 (14.3%)	34/93 (36.6%)	0.100^b^
Clinical pregnancy rate (%)	2/14 (14.3%)	30/93 (32.3%)	0.171^b^
Miscarriage rate (%)	0/14 (0%)	8/93 (8.6%)	0.202^c^
Implantation rate (%)	4/26 (15.38%)	34/183 (18.57%)	0.297^b^

a Independent student t test

b χ^2^- test

c Fisher’s exact test

**Table III:** Progesterone level ≥1.2 and pregnancy outcome correlation

	**Premature progesterone rise**	
	**Agonist**	**Antagonist**
Biochemical pregnancy rate	0.677	0.530
Clinical pregnancy rate	0.545	0.129
Miscarriage rate	0.383	0.087
Implantation rate	0.970	0.113

Spearman correlation coefficient.

**Table IV:** Progesterone level ≥1.5 and pregnancy outcome correlation

	**Premature progesterone rise**	
	**Agonist**	**Antagonist**
Biochemical pregnancy rate	0.834	0.822
Clinical pregnancy rate	0.754	0.589
Miscarriage rate	0.878	0.544
Implantation rate	0.536	0.545

Spearman correlation coefficient.

## Discussion

The subject of this research is not new but the issue is still unresolved (16). There are some studies about relationship between premature progesterone rise and pregnancy outcomes from 1991 (26). Schoolcraft *et al* concluded that progesterone concentrations of more than 0.5 ng/mL were associated with a significantly lower rate of pregnancy compared with those samples contained less than 0.5 ng/mL of progesterone that is in contrast with our results. This could be due to different understudied population or laboratory condition (26). 

In 1993, Check *et al* expressed significantly higher pregnancy rate in the group with p≤1 ng/ml at the time of hCG vs. the groups with the P level ranged from 1.1-2 ng/ml. In contrast to the previous related studies, check *et al* results showed no differences in groups with p<0.5 ng/ml versus 0.5-1 ng/ml (27). In 2012 Ochsenkuhn *et al* showed that a rise of serum p≥2.0 ng/mL on the day of hCG administration was associated with impaired early embryo implantation and reduced live birth rate in cycles with GnRH agonists after day-5 fresh ET. They concluded that the threshold for detrimental P levels should be individualized for each IVF institute because of the diversity of available P assays and duration of embryo culture (4).

Results from present study showed the women underwent COH with pituitary down-regulation by GnRH agonist and antagonist/ICSI had no significant lower clinical pregnancy rate in the case of a subtle P rise (p≥1.2) on the day of hCG administration. Some studies showed negative effects of elevated P levels on pregnancy outcome. Doldi *et al* showed elevated serum progesterone on the day of HCG administration in IVF is associated with a higher pregnancy rate in polycystic ovary syndrome but this prospective study did not reach to statistical significance, probably because of small sample size (8, 10, 12, 28, 29). In some studies, P levels distribution has evaluated on the same day of oocyte retrieval as it relates to pregnancy outcome in an antagonist, but our study was about evaluated P at HCG administration day (11). 

In one study, pregnancy rates after day-3 or day-5 embryo transfer were reported, and interestingly, even modest rises of P in the follicular phase have detrimental effect on the implantation potential of a good-quality cleavage stage embryo. On the contrary, premature luteinization in the blastocyst subgroup had no effect on the pregnancy outcome (30). In a different manner, we employed two or three days old embryos, and excluded blastocysts. Incidence of premature leutinization varies among different down regulatory protocols in COH; the frequency has been reported 5-35% in GnRH agonist cycles and 9-38% in GnRH antagonist cycles (4-8). In our study, serum levels of progesterone on day of hCG were ≥1.2 ng/mL in 14 cycles (13.08%), while values ≥1.5 ng/mL were found only in three cases (2.8%), the incidence of PPR (p≥1.2 ng/ml) in long agonist protocol was15.2% and in antagonist protocol was 11.5% and accordingly, no significant different was conducted in the two groups.

In this study, clinical and biochemical pregnancy rate and implantation rate were lower and miscarriage rate was higher in antagonist group than agonist group, probably because of higher range in age and infertility duration of patients in antagonist group. Moreover, correlation between ICSI outcome and the serum P was calculated for either 1.2 ng/ml or 1.5 ng/ml serum P cut off level and found no significant correlation between P levels and pregnancy outcome, other studies were found negative effects of PPR on pregnancy outcome in different threshold levels such as p=0.9 ng/mL, 1.0 ng/mL, 1.7 ng/mL and 1.99 ng/mL (4, 27, 28, 31). It seems be that the use of different progesterone cut-off levels leads to failure in observation of negative correlation between PPR and clinical pregnancy outcomes (1). 

These variable results could be owing to different sample number and different methodologies for hormone measurement, different patient populations or even human errors. In one study for assessment of PPR adverse effect on endometrial receptivity, they analyzed the outcome of frozen ET cycles and showed that live birth rates are not significantly different between the groups with or without PPR (1). In present study, we did not analyzed the outcome of frozen ET cycles because of different causes effect on the outcome of frozen ET cycles such as oocyte quality, embryo quality and laboratory techniques.

In summary our analysis on the pregnancy outcomes in 107 patients underwent ICSI cycles (agonist or antagonist) showed that PPR had not any correlation with clinical pregnancy rate, biochemical pregnancy rate, implantation rate, and miscarriage rate. Because of these conflicting findings, further larger prospective studies are required in the future to determine whether elevated P has correlation with lower pregnancy outcomes and to determine whether extended culture or embryo freezing is the preferred route for managing patients with elevated P.
